# Juvenile Delinquents

**Published:** 1920-12-25

**Authors:** 


### Juvenile Delinquents.

The Penal Reform League, whose aims have been sympathetically described in these columns, has obtained the co-operation of the State Children's Association in its efforts to promote the Bill for the better administration of the probation system. It is pointed out in a report issued by the Executive Committee of the latter Association, of which the Earl of Lytton is chairman, that the number of children and young persons brought before the Juvenile Courts of England and Wales during 1919 was 40,473, a decrease of over 9,000 on the figures for 1918. The decrease has been continuing since these figures were made available, so that there is ground for hope that they will soon fall at least to the pre-war figure of 37,000. The number of juvenile adults—that is, persons between sixteen and twenty-one—sent to prison during the same period was 4,079 lads and 1,098 girls, of whom 1,609 lads and 852 girls were committed for one month or under. The latter deplorable short-term commitments show an increase of 300 lads and 136 girls on the previous year's figures. Were these young people, it is pertinently asked, committed to prison in order that they might be reformed? The blighting and deteriorating influence of prison life on young and old has been proved beyond question. Were they sent there to "protect society?" They issue from the prison gates with minds seared and distorted, with spirits wounded, and rebellious against society. "A great many are not possessed of the real criminal instinct," reports a prison chaplain; why then do we treat them as criminals? There are indications of the growth of a much better informed attitude towards juvenile delinquents. We believe the time will soon come when that spirit will prevail also in the treatment of adolescents. The relation between crime and health—between mental and moral abnormality and physical abnormality—is often obscured, but it is very real. When close investigation has made the relationship plain and given it the true perspective, a better-informed public will insist on a humane reform of the penal code, yet one which avoids sickly sentimentality and the condonation of serious crime.

